# Understanding Medicare With Hearing Loss

**DOI:** 10.1093/geroni/igaa057.2891

**Published:** 2020-12-16

**Authors:** Amber Willink

**Affiliations:** The University of Sydney, Sydney, New South Wales, Australia

## Abstract

Medicare has become an increasingly complex program to navigate with numerous choices available to beneficiaries with important implications on their financial exposure and access to care. While research has identified poor health literacy as a barrier to understanding Medicare, little information is available on the experience of individuals with hearing loss. Using the Medicare Current Beneficiary Survey (2016), a nationally-representative sample of 10,841 beneficiaries, we examined if difficulty understanding Medicare was associated with reported trouble hearing, while controlling for socio-demographic and health literacy factors. Compared to no trouble, Medicare beneficiaries with a little or a lot of trouble hearing had 44% (95% CI OR:1.34-1.55) and 63% (95% CI OR: 1.44-1.83) increased odds of reporting greater difficulty with understanding Medicare. The existing tools to support Medicare beneficiaries understand and navigate the program must evolve to meet the needs of those with hearing loss- a highly prevalent condition among Medicare beneficiaries.

